# Increased Cytotoxicity of Herpes Simplex Virus Thymidine Kinase Expression in Human Induced Pluripotent Stem Cells

**DOI:** 10.3390/ijms20040810

**Published:** 2019-02-14

**Authors:** Chizuru Iwasawa, Ryota Tamura, Yuki Sugiura, Sadafumi Suzuki, Naoko Kuzumaki, Minoru Narita, Makoto Suematsu, Masaya Nakamura, Kazunari Yoshida, Masahiro Toda, Hideyuki Okano, Hiroyuki Miyoshi

**Affiliations:** 1 Department of Pharmacology, Hoshi University School of Pharmacy and Pharmaceutical Sciences, 2-4-41, Ebara, Shinagawa-ku, Tokyo 142-8501, Japan; d1501@hoshi.ac.jp (C.I.); n-kuzumaki@hoshi.ac.jp (N.K.); narita@hoshi.ac.jp (M.N.); 2 Department of Neurosurgery, Keio University School of Medicine, 35 Shinanomachi, Shinjuku-ku, Tokyo 160-8582, Japan; moltobello-r-610@hotmail.co.jp (R.T.); kazrmky@keio.jp (K.Y.); todam@keio.jp (M.T.); 3 Department of Biochemistry, Keio University School of Medicine, 35 Shinanomachi, Shinjuku-ku, Tokyo 160-8582, Japan; yuki.sgi@gmail.com (Y.S.); gasbiology@keio.jp (M.S.); 4 Department of Physiology, Keio University School of Medicine, 35 Shinanomachi, Shinjuku-ku, Tokyo 160-8582, Japan; s.suzuki@a7.keio.jp; 5 Department of Orthopedic Surgery, Keio University School of Medicine, 35 Shinanomachi, Shinjuku-ku, Tokyo 160-8582, Japan; masa@keio.jp

**Keywords:** herpes simplex virus type 1 thymidine kinase, induced pluripotent stem cells, lentiviral vector, genome editing, cytotoxic, nucleotide metabolism

## Abstract

Human induced pluripotent stem cells (iPSCs) hold enormous promise for regenerative medicine. The major safety concern is the tumorigenicity of transplanted cells derived from iPSCs. A potential solution would be to introduce a suicide gene into iPSCs as a safety switch. The herpes simplex virus type 1 thymidine kinase (*HSV-TK*) gene, in combination with ganciclovir, is the most widely used enzyme/prodrug suicide system from basic research to clinical applications. In the present study, we attempted to establish human iPSCs that stably expressed HSV-TK with either lentiviral vectors or CRISPR/Cas9-mediated genome editing. However, this task was difficult to achieve, because high-level and/or constitutive expression of HSV-TK resulted in the induction of cell death or silencing of HSV-TK expression. A nucleotide metabolism analysis suggested that excessive accumulation of thymidine triphosphate, caused by HSV-TK expression, resulted in an imbalance in the dNTP pools. This unbalanced state led to DNA synthesis inhibition and cell death in a process similar to a “thymidine block”, but more severe. We also demonstrated that the Tet-inducible system was a feasible solution for overcoming the cytotoxicity of HSV-TK expression. Our results provided a warning against using the *HSV-TK* gene in human iPSCs, particularly in clinical applications.

## 1. Introduction

Human somatic cells can be reprogrammed by transducing defined factors into pluripotent stem cells, termed induced pluripotent stem cells (iPSCs) [1,2]. Human iPSC technology has been widely used for disease modeling, drug discovery, and cell therapy development [3,4,5]. For applications in regenerative medicine, human iPSCs circumvent the ethical issues associated with human embryonic stem cells (ESCs). Moreover, autologous iPSCs or HLA-matched donor-derived iPSCs may overcome the problem of immune rejection. However, genetic and epigenetic abnormalities occur during reprogramming and prolonged growth in cell culture [6,7]. Consequently, a critical concern is the risk of tumorigenesis, particularly when using iPSC-derived dividing cells such as neural stem/progenitor cells (NS/PCs) [8,9].

Several strategies are currently being explored in an effort to reduce the potential tumorigenicity before the transplantation of iPSC-derived cells. These strategies include improving the quality of iPSCs and cell culture methods, selecting desired cell types with antibodies against specific cell surface markers, and using small-molecule inhibitors for the selective elimination of undifferentiated iPSCs in vitro or prevention of tumor-like overgrowth of iPSC-derived transplants [10,11,12,13,14]. However, it is difficult to eliminate the tumorigenicity of iPSC-derived cells completely. A more promising approach might be the implementation of safety-switch strategies with suicide genes, such as herpes simplex virus type 1 thymidine kinase (*HSV-TK*), cytosine deaminase, and inducible caspase-9. With these strategies, undesired tumorigenic iPSC-derived cells can be ablated following transplantation [15,16,17,18,19].

The most widely used enzyme/prodrug suicide system is the *HSV-TK* gene in combination with ganciclovir (GCV) [20,21]. Unlike cellular thymidine kinase, the HSV-TK exhibits broad substrate specificity; thus, HSV-TK phosphorylates non-toxic GCV, and cellular kinases further phosphorylate GCV to produce GCV triphosphate. GCV triphosphate inhibits DNA chain elongation, which induces apoptosis in dividing cells. Moreover, the phosphorylated GCV is able to pass through gap junctions to spread its cytotoxic effects to neighboring dividing cells that do not express HSV-TK, representing the so-called “bystander effect” [22,23]. Cell cycle-dependent apoptosis and the bystander effect of the HSV-TK/GCV system provide benefits, particularly in cancer treatments, including stem cell-based therapies [21,24,25,26].

In this study, we attempted to establish human iPSCs that stably expressed HSV-TK. This task was difficult to achieve, because high-level and/or constitutive HSV-TK expression was highly cytotoxic to human iPSCs. We also performed a metabolome analysis focused on nucleotides to elucidate how HSV-TK expression induced cytotoxicity in human iPSCs.

## 2. Results

### 2.1. Human iPSCs Transduced with Lentiviral Vectors Expressing HSV-TK

**Figure 1:**
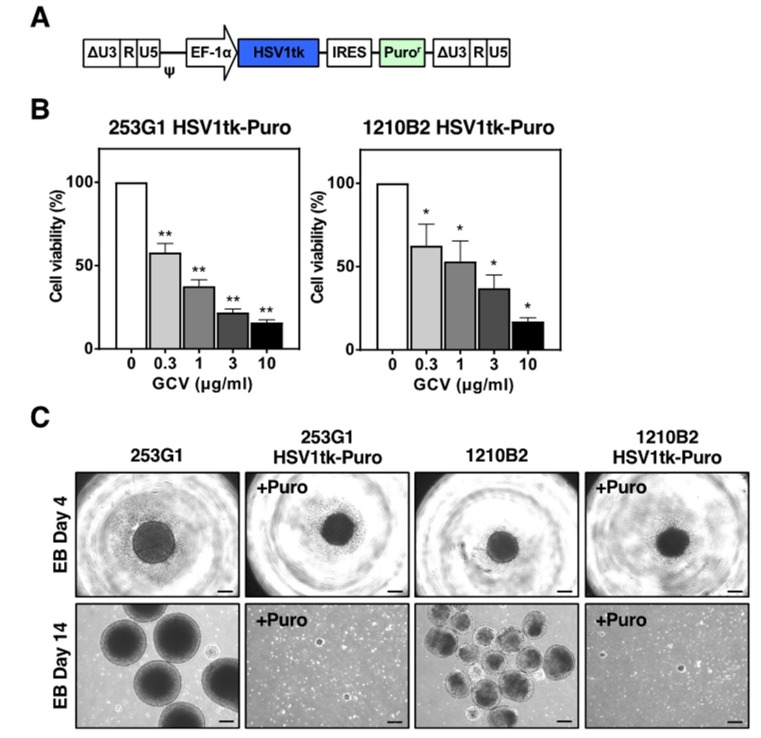
Transduction of human iPSCs with the lentiviral vector expressing the HSV-TK gene. (**A**) Schematic representation of the integrated proviral form of the lentiviral vector expressing the *HSV1tk* gene. HSV1tk, humanized-codons with CpG-free *HSV-TK* gene; EF-1α, human elongation factor 1 α subunit promoter; IRES, internal ribosomal entry site; Puro^r^, puromycin resistance gene; ΔU3, deletion of enhancer/promoter in the U3 region of the LTR; ψ, packaging signal. (**B**) Puromycin-resistant 253G1 and 1210B2 iPSCs transduced with the lentiviral vector expressing the *HSV1tk* gene were cultured in the presence of various concentrations of GCV for 2–5 days. Cell viability was assessed by the CCK-8 assay. The percent cell viability was calculated relative to cells in the absence of GCV. There was no significant difference in the results obtained on days 2, 3, 4, and 5 of culture. Data represent the mean ± SEM (*n* = 4–5). *, *p* < 0.05; **, *p* < 0.01. (**C**) Representative images of EB formation of 253G1, 1210B2, 253G1 HSV1tk-Puro, and 1210B2 HSV1tk-Puro iPSCs on day 4 and day 14. 253G1 HSV1tk-Puro and 1210B2 HSV1tk-Puro iPSCs were cultured with 1 μg/mL puromycin (+Puro). Scale bar, 200μm.

To establish human iPSCs that stably expressed HSV-TK, we transduced 253G1 and 1210B2 iPSCs with the lentiviral vector, CSII-EF-HSV1tk-IRES2-Puro. This vector contained the *HSV1tk* gene, which is the *HSV-TK* gene modified by humanizing the codon usage and eliminating the CpG motifs, and the puromycin resistance gene under the control of the human elongation factor 1 α subunit (EF-1α) promoter (Figure 1A). We chose the EF-1α promoter that confers high levels of transgene expression in iPSCs and NS/PCs, because we plan to use the HSV-TK/GCV system as a safety switch in iPSC-derived NS/PC transplantation for the treatment of spinal cord injury and as a suicide gene therapy for malignant glioma using iPSC-derived NS/PCs. iPSCs were transduced at a multiplicity of infection (MOI) of <1, because cell death occurred at high MOIs (>5). On the other hand, when we infected human iPSCs with the control vector, which only contained the Venus fluorescent protein gene [27], we observed ~100% transduction at MOIs of 5–10 with no cell death.

Transduced iPSCs were cultured under puromycin selection, and puromycin-resistant iPSCs were obtained at very low efficiency. Transduced cells grew slightly slower than non-transduced cells (doubling time: 17.05 ± 0.48 h (253G1) vs. 17.31 ± 1.39 h (253G1 HSV1tk-Puro) (*n* = 3); 14.54 ± 0.06 h (1210B2) vs. 23.3 ± 1.55 h (1210B2 HSV1tk-Puro) (*n* = 3)). Puromycin-resistant iPSCs showed a dose-dependent sensitivity to GCV (Figure 1B). Next, we cultured puromycin-resistant iPSCs to form embryoid bodies (EBs). However, iPSCs failed to form EBs under puromycin selection (Figure 1C). On the other hand, iPSCs could form EBs without puromycin selection, but the NS/PCs generated from these EBs were no longer resistant to puromycin or sensitive to GCV. Similar results were obtained with iPSCs transduced with the lentiviral vector, CSII-EF-HSV-TK-1-IRES2-Puro, which carried the original unmodified *HSV-TK* gene, *HSV-TK-1*. (Supplementary Figure S1). These results implied that high-level and/or constitutive expression of HSV-TK was cytotoxic in iPSCs, and transgene silencing might have occurred.

**Figure 2:**
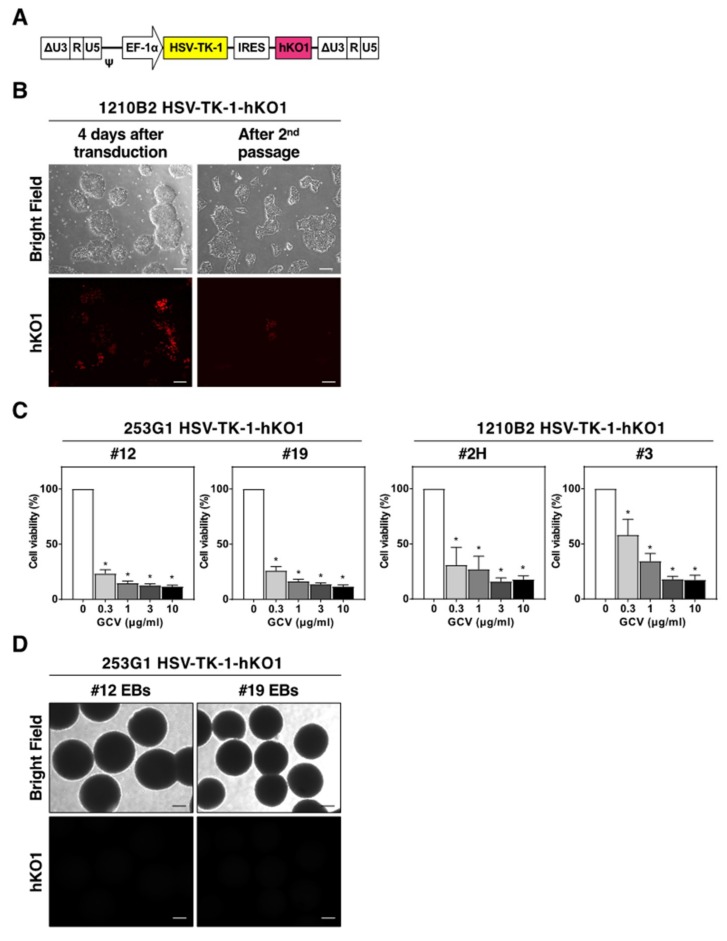
Silencing the transgene in human iPSCs. (**A**) Schematic representation of the integrated proviral form of the lentiviral vector expressing the *HSV-TK-1* gene. HSV-TK-1, original *HSV-TK* gene; hKO1, humanized-codon Kusabira-Orange fluorescent protein gene. (**B**) Representative images of 1210B2 iPSCs 4 days after lentiviral transduction and after the second passage. Scale bar, 200 μm. (**C**) hKO1-positive iPSC clones, 253G1 HSV-TK-1-hKO1 (#12, #19) and 1210B2 HSV-TK-1-hKO1 (#2H, #3), were cultured in the presence of various concentrations of GCV for 3 days. Cell viability was assessed by the CCK-8 assay. The percent cell viability was calculated relative to cells in the absence of GCV. Data represent the mean ± SEM (*n* = 4). *, *p* < 0.05. (**D**) Representative images of EB formation of 253G1 HSV-TK-1-hKO1 iPSCs (#12, #19) on day 14. hKO1 fluorescence signal was not detected. Scale bar, 200 μm.

To investigate the possibility of transgene silencing, we transduced 253G1 and 1210B2 iPSCs with the lentiviral vector, CSII-EF-HSV-TK-1-IRES2-hKO1, which carried the humanized-codon Kusabira-Orange (hKO1) fluorescent protein gene [28], at a MOI of ~0.5 (Figure 2A). Although 70–80% of iPSCs were hKO1-positive immediately after transduction, the proportion of hKO1-positive cells decreased with time, and <5% of iPSCs were hKO1-positive after the second passage (Figure 2B). Single iPSCs with high and medium hKO1 expression levels (hKO1^high^ and hKO1^med^, respectively) were sorted by fluorescence-activated cell sorting (FACS) and were individually cultured. However, <25% of iPSC clones could be expanded, and the hKO1^med^ iPSC population yielded more expanded clones than the hKO1^high^ population. We found that hKO1 expression was frequently silenced, and these clones became resistant to GCV. For some clones, the vast majority of cells were hKO1-positive but GCV resistant, which suggested that mutations in the *HSV-TK* gene occurred during lentiviral reverse transcription or after lentiviral integration. Only a few clones stably expressed hKO1 and displayed GCV sensitivity (Figure 2C, Supplementary Figure S2). However, when cultured to form EBs, these clones were unable to form EBs (e.g., 1210B2 HSV-TK-1-hKO1, clones #2H and #3) or they formed EBs with silenced hKO1 expression (e.g., 253G1 HSV-TK-1-hKO1, clones #12 and #19) (Figure 2D). This result suggested that HSV-TK expression might be more cytotoxic to EBs than to iPSCs, due to the higher cell density of EBs. Similar results were obtained with the lentiviral vector that carried the human ubiquitin C promoter, a weak promoter of HSV-TK expression, compared to the EF-1α promoter, in iPSCs. On the other hand, when we transduced U87 human glioblastoma cells with the lentiviral vector, CSII-EF-HSV1tk-IRES2-hKO1, FACS-sorted hKO1^high^ populations could be expanded without silencing of the hKO1 expression. The hKO1-positive U87 cells remained sensitive to GCV (Supplementary Figure S3), though the growth rate was slightly slower than that of non-transduced U87 cells.

It was previously reported that a cryptic promoter, located between the first and second ATG codons of the *HSV-TK* gene, led to ectopic HSV-TK expression in the testis of transgenic mice and rats, which caused male sterility [29,30,31]. Transgenic mice carrying a truncated *HSV-TK* gene lacking the cryptic promoter, which retained functional kinase activity comparable to that of intact HSV-TK, were shown to abolish male sterility [32]. It was unlikely that the cryptic promoter was involved in the cytotoxicity of high-level HSV-TK expression in iPSCs, because we obtained similar results with either the *HSV1tk* gene or the *HSV-TK-1* gene, despite the 75% nucleotide sequence homology between these genes. Nonetheless, in subsequent experiments, we used the lentiviral vector CSII-EF-del-HSV-TK-1-IRES2-hKO1, which contained a truncated version of the *HSV-TK* gene, *del-HSV-TK-1*, to transduce 253G1 and 1210B2 iPSCs. Our results showed that most iPSCs could not survive at high MOIs (>1) and that hKO1 expression was rapidly silenced in surviving iPSCs.

### 2.2. Targeted Insertion of the HSV-TK Gene into the GAPDH Locus with CRISPR/Cas9-Mediated Genome Editing

Lentiviral vectors integrate randomly into the host genome and are prone to transgene silencing via position effects. Therefore, CRISPR/Cas9-mediated genome editing was used to insert the *HSV-TK* gene into the glyceraldehyde-3-phosphate dehydrogenase (*GAPDH*) housekeeping gene locus in 253G1, 1210B2, and 1231A3 iPSCs, so that the *HSV-TK* gene could be consistently expressed by the endogenous GAPDH promoter (Supplementary Figure S4). Using the homologous recombination (HR) donor plasmid, HR-GAPDH-2A-HSV1tk-2A-Venus, which is designed to insert the *HSV1tk* gene, flanked by a self-cleaving 2A peptide sequence, followed by the *Venus* gene, before the stop codon of *GAPDH* gene and express the *GAPDH-HSV1tk-Venus* fusion gene, the initial frequency of Venus-positive colonies (~0.1%) was similar to that observed with the control HR donor plasmid, HR-GAPDH-2A-Venus. However, after the first passage, Venus-positive colonies were rarely observed. Single, 10, or 100 Venus-positive iPSCs were sorted by FACS and cultured in 96-well plates. Most Venus-positive iPSCs were unable to expand, and all surviving colonies were Venus-negative (Table 1). Similar results were obtained with the HR-GAPDH-2A-HSV-TK-1-2A-Venus donor plasmid. In contrast, with the control HR-GAPDH-2A-Venus plasmid, >10% of FACS-sorted Venus-positive iPSCs survived, and silencing of Venus expression did not occur in these expanded clones.

Next, we used the HR-GAPDH-2A-HSV1tk-2A-Puro donor plasmid to select puromycin-resistant iPSCs (Supplementary Figure S4). Only a few puromycin-resistant colonies were obtained from 2 × 10^6^ transfected iPSCs, and they were all GCV resistant (Table 2). Genomic PCR analysis of individual puromycin-resistant clones indicated that the *HSV1tk-Puro* fusion gene cassette was not correctly integrated into the *GAPDH* gene locus. In contrast, >20 puromycin-resistant clones were obtained from 1 × 10^6^ transfected HeLa cells, and a genomic PCR analysis demonstrated correct integration in some GCV-sensitive clones.

### 2.3. Cytotoxicity of HSV-TK Expression Investigated with Tet-Inducible Lentiviral Vectors

**Figure 3:**
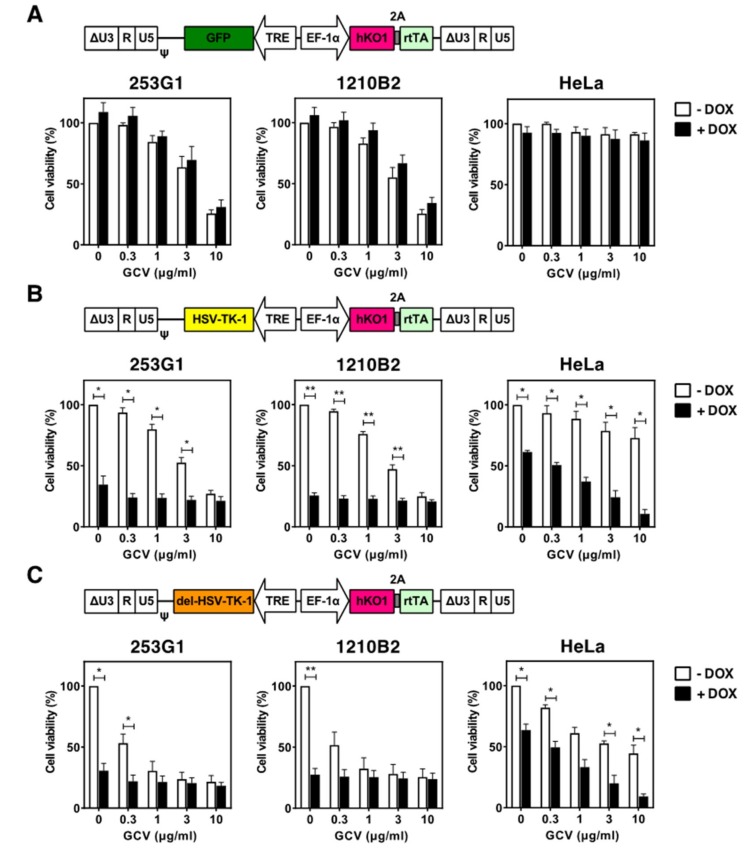
Cytotoxicity of HSV-TK expression with Tet-inducible lentiviral vectors. (**A**–**C**) Schematic representations of the integrated proviral forms of the Tet-inducible lentiviral vectors, which carried (**A**) the *GFP* gene, (**B**) the *HSV-TK-1* gene, and (**C**) the *del-HSV-TK-1* gene. del-HSV-TK-1, truncated *HSV-TK* gene without the cryptic promoter; TRE, Tet-responsive promoter; rtTA; reverse Tet-controlled transactivator protein gene. 253G1 iPSCs, 1210B2 iPSCs, and HeLa cells transduced with the indicated Tet-inducible lentiviral vectors were cultured in the presence of various concentrations of GCV, with or without 1 μg/mL doxycycline (Dox) for 3–4 days. Cell viability was assessed by the CCK-8 assay. The percent cell viability was calculated relative to cells in the absence of GCV without Dox. There was no significant difference in the results obtained on days 3 and 4 of culture. Data represent the mean ± SEM (*n* = 4–6). *, *p* < 0.05; **, *p* < 0.01.

To investigate the cytotoxicity of HSV-TK expression further, we infected 253G1 and 1210B2 iPSCs and HeLa cells with tetracycline (Tet)-inducible lentiviral vectors that contained the *HSV-TK-1*, *del-HSV-TK-1*, *HSV1tk*, or green fluorescent protein (*GFP*) gene (Figure 3, Supplementary Figure S5). The hKO1^high^ cell population was sorted by FACS and expanded. In these cells, hKO1 expression was rarely silenced. In a control experiment with the Tet-inducible GFP vector, no cytotoxicity was observed with doxycycline (Dox) treatment in iPSCs or HeLa cells, but iPSCs exhibited a higher sensitivity to GCV (Figure 3A). On the other hand, with the Tet-inducible HSV-TK-1, del HSV-TK-1, and HSV1tk vectors, HSV-TK expression was cytotoxic in iPSCs upon Dox treatment in the absence of GCV. The percentage of cell death was nearly comparable to that observed in the presence of GCV (Figure 3B,C, Supplementary Figure S5). Similar results were obtained when the other iPSC line (201B7) was transduced with the Tet-inducible HSV1tk vector. Cytotoxicity, albeit to a lesser extent, was also observed in HeLa cells in the absence of GCV (Figure 3B,C, Supplementary Figure S6). Cell cycle analysis showed a significant increase in the percentage of cells in the S phase with HSV-TK expression (Supplementary Figure S7).

Several clones were established from 253G1 and 1210B2 iPSCs transduced with the Tet-inducible HSV1tk vector. These clones were able to form EBs and differentiate into NS/PCs, which were sensitive to GCV (Supplementary Figure S8). Note that cytotoxicity was not significantly observed in NS/PCs upon Dox treatment in the absence of GCV. These results demonstrated that HSV-TK expression-induced cytotoxicity was more severe in iPSCs than in NS/PCs, as well as in HeLa cells.

### 2.4. Nucleotide Metabolism in iPSCs That Expressed HSV-TK

**Figure 4:**
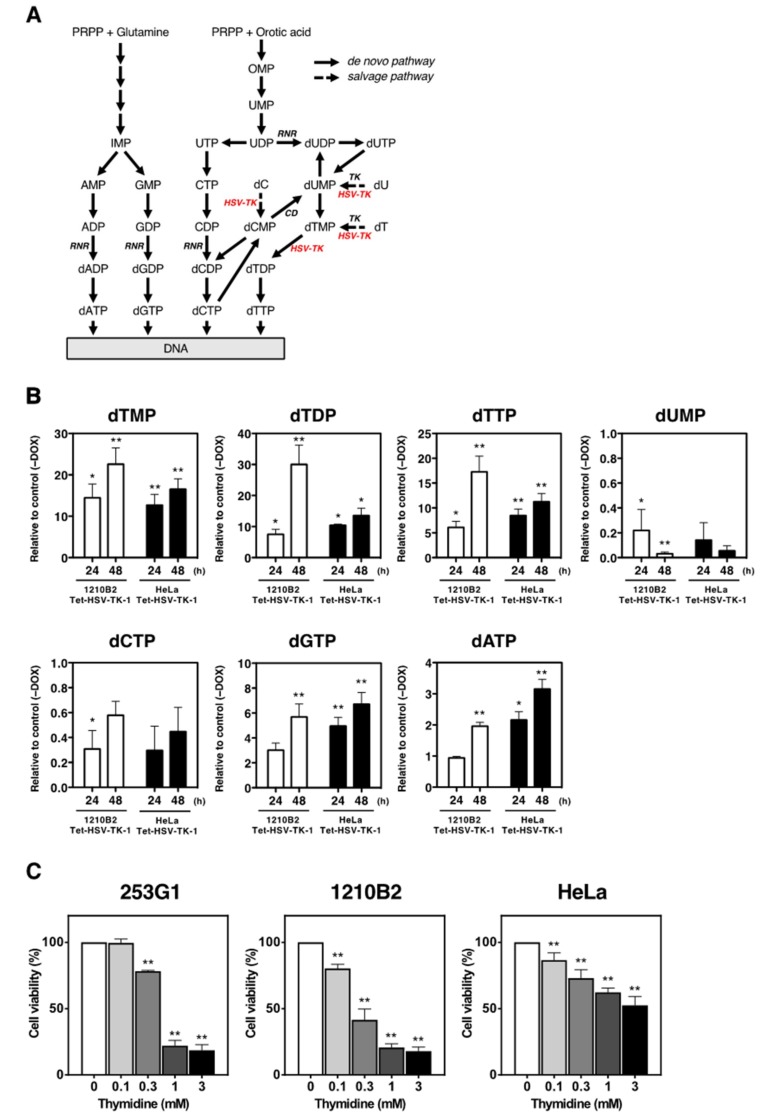
Analysis of nucleotide metabolism in human iPSCs expressing HSV-TK and the effect of excess dT on cell viability. (**A**) Schematic diagram of the nucleotide synthesis pathway. Solid arrows indicate the de novo pathway. Dashed arrows indicate the salvage pathway. HSV-TK (red) phosphorylates dT, dU, dC, and dTMP. PRPP, 5-phospho-α-d-ribosyl-1-pyrophosphate; RNR, ribonucleotide reductase; TK, thymidine kinase; CD, dCMP deaminase. (**B**) 1210B2 iPSCs and HeLa cells transduced with the Tet-inducible lentiviral vector carrying the *HSV-TK-1* gene were cultured with or without 1 μg/mL Dox. Metabolome analysis of the indicated nucleotides was performed at the indicated time points. Data are expressed as the fold change in nucleotide levels relative to corresponding cells without Dox (control). Data represent the mean ± SEM (*n* = 4–5). *, *p* < 0.05; **, *p* < 0.01. (**C**) 253G1 iPSCs, 1210B2 iPSCs, and HeLa cells were cultured in the presence of various concentrations of dT for 2–3 days. Cell viability was assessed by the CCK-8 assay. The percent cell viability was calculated relative to cells in the absence of dT. There was no significant difference in the results obtained on days 2 and 3 of culture. Data represent the mean ± SEM (*n* = 5–6). **, *p* < 0.01.

Unlike the cellular thymidine kinase, HSV-TK phosphorylates not only thymidine (dT) and deoxyuridine (dU), but also deoxycytidine (dC) and thymidine monophosphate (dTMP) (Figure 4A). To investigate why HSV-TK expression was highly cytotoxic to iPSCs, we analyzed nucleotide metabolism in iPSCs and HeLa cells transduced with the Tet-inducible HSV-TK-1 vector. After Dox treatment, the intracellular levels of dTMP, thymidine diphosphate (dTDP), and thymidine triphosphate (dTTP) were increased in both 1210B2 iPSCs and HeLa cells as the result of HSV-TK expression (Figure 4B). As expected, the accumulation of dTTP led to the inhibition of dCMP deaminase (CD), which regulates the dCTP/dTTP ratio, resulting in a conspicuous decrease in dUMP levels (Figure 4A,B). In addition, the accumulation of dTTP is assumed to activate the reduction of GDP to dGDP catalyzed by ribonucleotide reductase (RNR), a key allosteric enzyme in regulating deoxyribonucleoside triphosphate (dNTP) levels [33,34], leading to the consequent formation of dGTP (Figure 4A). The accumulation of dGTP, in turn, activates the reduction of ADP to dADP followed by the formation of dATP, which then inhibits the reduction of all substrates by RNR. In HeLa cells, we observed increased levels of dGTP and dATP, which were thought to be allosterically regulated by RNR; however, the dTTP levels were increased to a much greater extent compared to the increases observed in dGTP and dATP levels, while dCTP levels were decreased (Figure 4B). Similarly, in iPSCs, the levels of dTTP, dGTP, and dATP were increased and the dCTP levels were deceased. However, the increase in dTTP levels was markedly higher and the increase in dATP levels was lower, when compared with HeLa cells (Figure 4B). Thus, HSV-TK expression affected the balance of the dNTP pools more in iPSCs than in HeLa cells. On the other hand, the levels of ribonucleoside triphosphates (NTPs) were not altered substantially with HSV-TK expression in both iPSCs and HeLa cells (Supplementary Figure S9). These results suggested that the accumulation of dTTP caused an imbalance in the dNTP pools, which led to DNA synthesis inhibition and cell death. This inhibition of DNA synthesis was reminiscent of that observed in cells treated with excess dT, a procedure for synchronizing cells (known as a ‘thymidine block’) [35].

Next, we examined the effect of dT on cell viability. The addition of 1–3 mM dT to cell cultures, a concentration commonly used for a thymidine block, significantly reduced cell viability in HeLa cells. Moreover, the sensitivity to dT was higher in iPSCs than in HeLa cells (Figure 4C), consistent with the cytotoxic effect of HSV-TK expression. Taken together, our data demonstrated that HSV-TK expression resulted in imbalanced dNTP pools through the accumulation of dTTP, which led to DNA synthesis inhibition, in a process similar to a thymidine block. Moreover, this effect was more severe in iPSCs than in HeLa cells.

## 3. Discussion

The HSV-TK/GCV suicide system has been widely used for cancer gene therapy. In mouse ESC gene targeting, the *HSV-TK* gene is a frequently used as a negative selection marker to eliminate non-homologous insertions. In these applications, the stable expression of HSV-TK is not required. In the present study, we found it difficult to establish human iPSCs that stably expressed HSV-TK, because the high-level and/or constitutive expression of HSV-TK resulted in either cell death or HSV-TK silencing. To our knowledge, only three previous studies to date have reported the successful expression of HSV-TK in human PSCs [16,36,37]. Of those studies, two used ESCs that expressed the *HSV-TK* gene under the control of either the phosphoglycerate kinase (PGK) promoter by plasmid transfection or the cell cycle-dependent Ki67 promoter by lentiviral transduction (transduction efficiency: 10–20%), and the neomycin or zeocin resistance gene was used for the selection of transduced cells [36,37]. Only one study used both ESCs and iPSCs in which the *HSV-TK* gene was driven by the EF-1α or Nanog promoter and the puromycin resistance gene was driven by the PGK promoter in the lentiviral vectors and a high MOI (=50) was used for transduction [16]. Although the expression levels of HSV-TK in these previous studies were not measured and could not be compared with those in the present study, there may be optimal expression levels of HSV-TK in human PSCs.

The cytotoxic effect of HSV-TK expression was previously described in transgenic mice and rats, where ectopic high-level expression of HSV-TK in the testis caused abnormal spermatogenesis and male sterility [29,30,31]. In those studies, it was speculated that the kinase activity of highly expressed HSV-TK might consume a large amount of ATP, which could disturb sperm development. iPSCs could also be susceptible to a high consumption of ATP, given that PSCs, such as ESCs and iPSCs, are characterized by a rapid proliferation and low ATP content [38,39,40]. However, our results showed that HSV-TK expression did not substantially affect ATP levels (Supplementary Figure S9). 

Our analysis of nucleotide metabolism demonstrated that HSV-TK expression caused an excessive accumulation of dTTP, resulting in an imbalance in the dNTP pools. Maintaining appropriate dNTP levels is critical for fidelity in DNA replication and repair [41]. dNTPs can be generated through the de novo and salvage pathways. Although the de novo pathway dominates in cycling cells, the salvage pathway disturbed by the exogenous expression of HSV-TK may have cytotoxic effects. RNR and CD play central roles in the de novo synthesis of dNTPs (Figure 4A) [33,34,41]. Ordinarily, when dTTP levels are increased, CD is inhibited, leading to an increase in dCTP levels. RNR is simultaneously activated to reduce GDP to dGDP, thus increasing dGTP levels, which in turn activates ADP reduction to dADP, with consequent increasing dATP levels. Finally, high dATP/ATP ratios act to inhibit the activity of RNR [42]. These mechanisms maintain a balance among all four dNTP levels. However, with a thymidine block, the increase of dTTP levels is uncontrolled by the normal regulatory process and leads to the depletion of dCTP pools through the allosteric inhibition of RNR, resulting in an accumulation of cells in the S phase of the cell cycle [35,41]. Therefore, it is conceivable that excessive dTTP accumulation caused by HSV-TK expression might have similarly, but more severely, inhibited DNA synthesis.

Human iPSCs showed an increased cytotoxicity of HSV-TK expression and a higher sensitivity to dT compared to HeLa cells. This might be explained by a difference in nucleotide metabolism. Cultured PSCs have a peculiar cell cycle with a very short G_1_ phase, and they divide rapidly, even compared to cancer cells (Supplementary Figure S7) [43]. Rapidly dividing cells require a large amount of energy, and therefore, they must upregulate nucleotide synthesis. In this context, PSCs rely primarily on glycolysis for energy production [40,44]. While glycolysis is less efficient than oxidative phosphorylation (OXPHOS) in terms of ATP production, ATP can be generated quickly through glycolysis. Glycolytic intermediates enter the pentose phosphate pathway and serve as substrates for nucleotide synthesis. PSCs can be classified into two distinct pluripotent states: naïve and primed [45]. Mouse ESCs and iPSCs exhibit the naïve state, which corresponds to the pre-implantation stage of an embryo. Human ESCs and iPSCs and mouse epiblast stem cells exhibit the primed state, which corresponds to the post-implantation stage. Thus, primed PSCs represent a more differentiated state than naïve PSCs. Although glycolysis is predominant in both naïve and primed PSCs, naïve PSCs display bivalent metabolism utilizing both glycolysis and OXPHOS, whereas primed PSCs rely almost exclusively on glycolysis [40,46,47]. Glycolysis-skewed metabolism is also characteristic of highly proliferative cancer cells, where it is known as the Warburg effect [47]. However, cancer cells exhibit heterogeneity in energy metabolism and OXPHOS remains important in various cancer cell lines [48,49]. In HeLa cells, glutamine, not sugar, is the major energy source and thus OXPHOS provides most of the ATP under normal culture conditions [50,51]. Future metabolome analyses may elucidate causal processes involved in the cytotoxic effect of HSV-TK expression in human iPSCs.

Our results provided a warning against the use of HSV-TK in regenerative medicine or gene therapy with human iPSCs. For example, even if human iPSCs stably expressing HSV-TK are established, HSV-TK expression might disturb nucleotide metabolism and alter stemness. Silencing of HSV-TK expression may occur during NS/PC differentiation, resulting in an insufficient therapeutic effect. However, we demonstrated that the Tet-inducible system was a feasible solution to overcome the cytotoxicity of HSV-TK expression, although this system required both DOX and GCV for the induction of cell death. Indeed, we have recently shown that the Tet-inducible HSV-TK expression with GCV administration successfully ablated immature proliferative cells while preserving mature differentiated neurons following iPSC-derived NS/PC transplantation into the injured spinal cords of immune-deficient mice [19].

## 4. Materials and Methods 

### 4.1. Cell Culture

Human iPSCs (253G1 [52], 1210B2 [53], 1231A3 [54], and 201B7 [1]) (kindly provided by Shinya Yamanaka, Kyoto Univ., Kyoto, Japan) were cultured with a feeder-free protocol [53]. EB formation and NS/PC generation were performed as described previously [54]. HeLa and U87 cells were cultured in Dulbecco’s Modified Eagle’s Medium (Sigma-Aldrich, St. Louis, MO, USA) with 10% fetal bovine serum.

### 4.2. Lentiviral Vector Preparation

The HSV1tk cDNA that had been modified by humanizing the codon usage and eliminating all CpG dinucleotides was purchased from InvivoGen (San Diego, CA, USA) and was amplified by PCR. The HSV-TK-1 cDNA was PCR-amplified from the pDNsam-HSV-TK plasmid (kindly provided by Shin Kaneko, Kyoto University). The del-HSV-TK-1 cDNA, a truncated version of HSV-TK-1, was PCR-amplified from the pMCS-AAT-PB:PGKpuroΔtk-derived plasmid [55]. All PCR-amplified HSV-TK cDNAs were cloned into the pENTR/D-TOPO entry vector plasmid (Thermo Fisher Scientific, Waltham, MA, USA), and the final vector sequences were verified by DNA sequencing. All HSV-TK cDNAs were then transferred to three lentiviral vector plasmids, CSII-EF-RfA-IRES2-Puro, CSII-EF-RfA-IRES2-hKO1, and CSIV-RfA-TRE-EF-KT [56], with the Gateway LR clonase (Thermo Fisher Scientific). All plasmids are available from Addgene (Available online: https://www.addgene.org). Recombinant lentiviral vector production and titer determination were performed as described previously [57].

### 4.3. CRISPR/Cas9-Mediated Genome Editing

The Cas9/sgRNA expression plasmid, pU6-GAPDHgRNA4-Cas9, was constructed by cloning DNA oligonucleotides coding for sgRNA targeting the stop codon of the *GAPDH* gene (5′-CCTCCAAGGAGTAAGACCCC-3′, the stop codon is underlined) into the *Bbs*I site of the pX330-U6-Chimeric_BB-CBh-hSpCas9 plasmid (Addgene plasmid #42230) [58]. To construct HR donor plasmids (Supplementary Figure S4), 1-kb fragments of the left and right homology arms of the *GAPDH* gene (without the TAA stop codon) were PCR-amplified from genomic DNA isolated from human fibroblasts, NB1RGB (RIKEN BRC, Ibaraki, Japan), and these fragments were cloned into the PrecisionX HR donor vector HR100PA-1 (System Biosciences, Palo Alto, CA, USA). Then, polycistronic cassettes containing the *HSV1tk* and *Venus* fusion gene, the *HSV1tk* and puromycin resistance fusion gene, or the *Venus* gene alone were inserted between the left and right homology arms, resulting in the HR donor plasmids HR-GAPDH-2A-HSV1tk-2A-Venus, HR-GAPDH-2A-HSV1tk-2A-Puro, and HR-GAPDH-2A-Venus. These HR donor plasmids were designed to be in frame with the C-terminus of GAPDH and express the fusion proteins joined with a self-cleaving 2A peptide sequence. All plasmids were verified by DNA sequencing.

For transfection, 1 × 10^6^ iPSCs or HeLa cells suspended in 100 μL Opti-MEM (Thermo Fisher Scientific) were mixed with the pU6-GAPDHgRNA4-Cas9 plasmid (3 μg) and the HR donor plasmid (10 μg) and were subjected to electroporation at 125 V for 5 ms using a NEPA21 electroporator (Nepa Gene, Chiba, Japan). Immediately after electroporation, cells were plated in complete medium and were then subjected to FACS-sorting or puromycin selection (0.5 μg/mL).

To verify integration, genomic PCR analysis was performed with the following PCR primers:

GAPDH-GF1, 5′-CTTCTCTGCTGTAGGCTCATTTG-3′;
GAPDH-GR1, 5′- AACTCCTGACCTCAGGTGATACA-3′;
GAPDH-F1, 5′-CTAGGTATGACAACGAATTTGGC-3′;
GAPDH-R1, 5′-TGGTTGAGCACAGGGTACTTTAT-3′;
HSV1tk-R1, 5′-GTCTTAGGGCAGTTCTCCTGTTG-3′;
Puro-GF1, 5′-GAGCTGCAAGAACTCTTCCTCAC-3′;
Venus-GF1, 5′-ACAACCACTACCTGAGCTACCAG-3′;
Venus-GR1, 5′-GTAGTTGTACTCCAGCTTGTGCC-3′.

### 4.4. Cell Viability Assay

Cells were seeded in 96-well plates at a density of 5 × 10^3^ cells/200 μL/well. After 2–5 days of incubation, the cell viability assay was performed using the Cell Counting Kit-8 (CCK-8) (Dojindo Molecular Technologies, Kumamoto, Japan), which is based on detecting dehydrogenase activity in viable cells with a water-soluble tetrazolium salt (WST-8). The WST-8 formazan dye produced by viable cells was quantified by measuring the absorbance at 450 nm on an iMark Microplate Absorbance Reader (Bio-Rad, Hercules, CA, USA). Each experiment was performed in triplicate.

### 4.5. Metabolome Analysis

Metabolite extraction from cultured cells for metabolome analyses was performed as described previously [59]. Cells were washed twice with cold PBS, and residual wash solvent was carefully removed. The cell culture dishes were then immediately frozen by immersion into liquid nitrogen and stored at −80 °C until assayed. The frozen cultured cells were scraped with methanol containing internal standards (IS, described below) (500 μL), followed by the addition of an equal volume of chloroform and 0.4 volumes of ultrapure water (LC/MS grade; Wako, Osaka, Japan). After centrifugation (3 cycles at 4000 rpm for 60 s), the aqueous phase was ultrafiltered using an ultrafiltration tube (Ultrafree MC-PLHCC; Human Metabolome Technologies, Inc., Yamagata, Japan). Then, the filtrate was concentrated with a vacuum concentrator. The concentrated filtrate was dissolved in 50 μL of ultrapure water and used for LC-MS/MS and IC-MS analyses.

2-morpholinoethanesulfonic acid and 1,3,5-benzenetricarboxylic acid were used as internal standards for anionic metabolites. These compounds are not present in the tissues; thus, they serve as ideal standards. Loss of endogenous metabolites during sample preparation was corrected by calculating the recovery rate (%) of the standards in each sample measurement. For metabolome analysis focused on nucleotides, anionic metabolites were measured using an orbitrap-type MS (Q-Exactive focus; Thermo Fisher Scientific) connected to a high performance ion-chromatography (IC) system (ICS-5000+, Thermo Fisher Scientific) that enables us to perform highly selective and sensitive metabolite quantifications owing to the IC-separation and Fourier Transfer MS principle. The IC was equipped with an anion electrolytic suppressor (Thermo Scientific Dionex AERS 500) to convert the potassium hydroxide gradient into pure water before the sample entered the mass spectrometer. The separation was performed using a Thermo Scientific Dionex IonPac AS11-HC, 4-μm particle size column. The IC flow rate was 0.25 mL/min supplemented post-column with 0.18 mL/min makeup flow of MeOH. The potassium hydroxide gradient conditions for IC separation were as follows: from 1 mM to 100 mM (0–40 min), 100 mM (40–50 min), and 1 mM (50.1–60 min), at a column temperature of 30 °C. The Q Exactive focus mass spectrometer was operated under an ESI negative mode for all detections. Full mass scan (*m*/*z* 70−900) was performed at a resolution of 70,000. The automatic gain control target was set at 3 × 10^6^ ions, and the maximum ion injection time was 100 msec. Source ionization parameters were optimized with the spray voltage at 3 kV, and other parameters were as follows: transfer temperature = 320 °C, S-Lens level = 50, heater temperature = 300 °C, Sheath gas = 36, and Aux gas = 10.

### 4.6. Statistical Analysis

Statistical analyses were performed with the nonparametric Mann-Whitney U test using GraphPad Prism (GraphPad Software, San Diego, CA, USA). A *p*-value of <0.05 was considered statistically significant.

## Figures and Tables

**Table 1:** Efficiency of targeted insertion of the *HSV1tk-Venus* fusion gene into the *GAPDH* locus with CRISPR/Cas9-mediated genome editing.

Human iPSCs	No. of FACS-Sorted Venus-Positive Cells/Well		
	1	10	100
	(No. of Surviving Cells/Total No. of Sorted Cells)		
**253G1**	0/26	0/10	1*/100
**1231A3**	1*/26	0/10	1*/100
**1210B2**	0/26	0/10	1*/200
**1210B2 (Cont.)**	6/24	2/20	20/200

Human iPSCs (253G1, 1231A3, and 1210B2) were subjected to transfection with the pU6-GAPDHgRNA4-Cas9 plasmid and the HR-GAPDH-2A-HSV1tk-2A-Venus donor plasmid. The indicated numbers of Venus-positive cells were FACS-sorted into individual wells of a 96-well plate. The numbers of surviving cells per the total number of FACS-sorted cells are shown. *, Venus negative cells; Cont., HR-GAPDH-2A-Venus donor plasmid was used as a control.

**Table 2:** Efficiency of targeted insertion of the *HSV1tk-Puro* fusion gene into the *GAPDH* locus with CRISPR/Cas9-mediated genome editing.

Human Cells	No. of Puro^r^ Colonies	
	Exp. 1	Exp. 2
**253G1**	1	0
**1231A3**	1	0
**1210B2**	1	2
**HeLa**	>20	ND

Human iPSCs (253G1, 1231A3, and 1210B2) and HeLa cells were subjected to transfection with the pU6-GAPDHgRNA4-Cas9 plasmid and the HR-GAPDH-2A-HSV1tk-2A-Puro donor plasmid. Each experiment (Exp.) was performed with 1 × 10^6^ cells. The numbers of puromycin-resistant (Puro^r^) colonies are shown. ND, not done.

## Supplementary Materials

Supplementary materials can be found at http://www.mdpi.com/1422-0067/20/4/810/s1.

## Abbreviations

iPSC	induced pluripotent stem cell
ESC	embryonic stem cell
NS/PC	neural stem/progenitor cell
HSV-TK	herpes simplex virus type 1 thymidine kinase
GCV	gancicrovir
EF-1α	elongation factor 1 α subunit
MOI	multiplicity of infection
hKO1	humanized-codon Kusabira-Orange
FACS	fluorescence-activated cell sorting
GAPDH	glyceraldehyde-3-phosphate dehydrogenase
HR	homologous recombination
Tet	tetracycline
GFP	green fluorescent protein
Dox	doxycycline
dT	thymidine
dU	deoxyuridine
dC	deoxycytidine
dTMP	thymidine monophosphate
dTDP	thymidine diphosphate
dTTP	thymidine triphosphate
CD	dCMP deaminase
RNR	ribonucleotide reductase
dNTP	deoxyribonucleoside triphosphate
NTP	ribonucleoside triphosphate
PGK	phosphoglycerate kinase
OXPHOS	oxidative phosphorylation
CCK-8	Cell Counting Kit-8
WST-8	water-soluble tetrazolium salt
IC	ion-chromatography
