# Supplementary material for: Increased Cytotoxicity of Herpes Simplex Virus Thymidine Kinase Expression in Human Induced Pluripotent Stem Cells
Source: Int J Mol Sci. 2019 Feb 14;20(4):810. doi: 10.3390/ijms20040810 (PMC6413063; doi:10.3390/ijms20040810)
Supplement: Supplementary file 1 [file ijms-20-00810-s001.pdf]

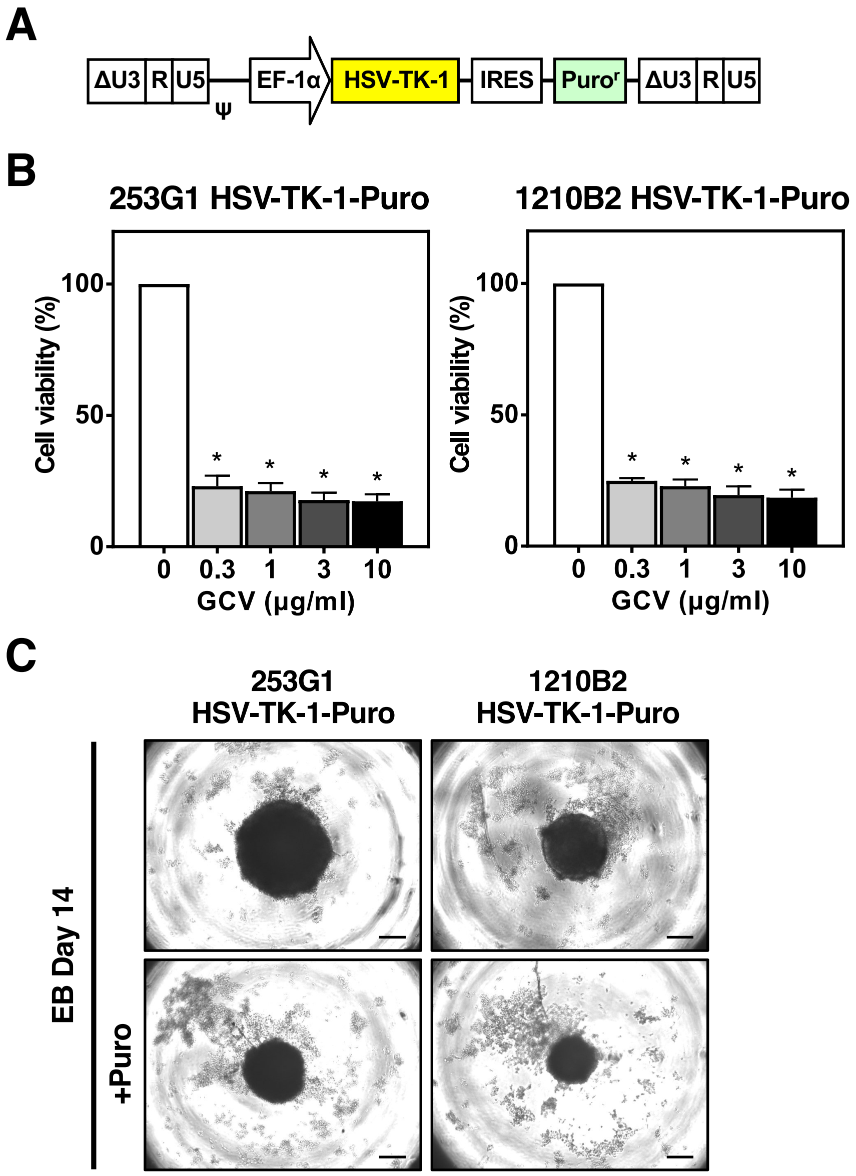

Supplementary Figure 1. Transduction of human iPSCs with the lentiviral vector expressing the *HSV-TK* gene. (**A**) Schematic representation of the integrated proviral form of the lentiviral vector expressing the *HSV-TK-1* gene. HSV-TK-1, original *HSV-TK* gene; EF-1α, human elongation factor 1 α subunit promoter; IRES; internal ribosomal entry site; Puro^r, puromycin resistance gene; ΔU3, deletion of enhancer/promoter in the U3 region of the LTR; ψ, packaging signal. (**B**) Puromycin-resistant 253G1 and 1210B2 iPSCs transduced with the lentiviral vector expressing the *HSV-TK-1* gene were cultured in the presence of various concentrations of GCV for 2–4 days. Cell viability was assessed by the CCK-8 assay. The percent cell viability was calculated relative to cells in the absence of GCV. There was no significant difference in the results obtained on days 2, 3, and 4 of culture. Data represent the mean ± SEM (n = 4). *, p <0.05. (**C**) Representative images of EB formation of 253G1 HSV-TK-1-Puro and 1210B2 HSV-TK-1-Puro iPSCs on day 14 cultured with or without 1 μg/ml puromycin (+Puro). Scale bar, 200μm.

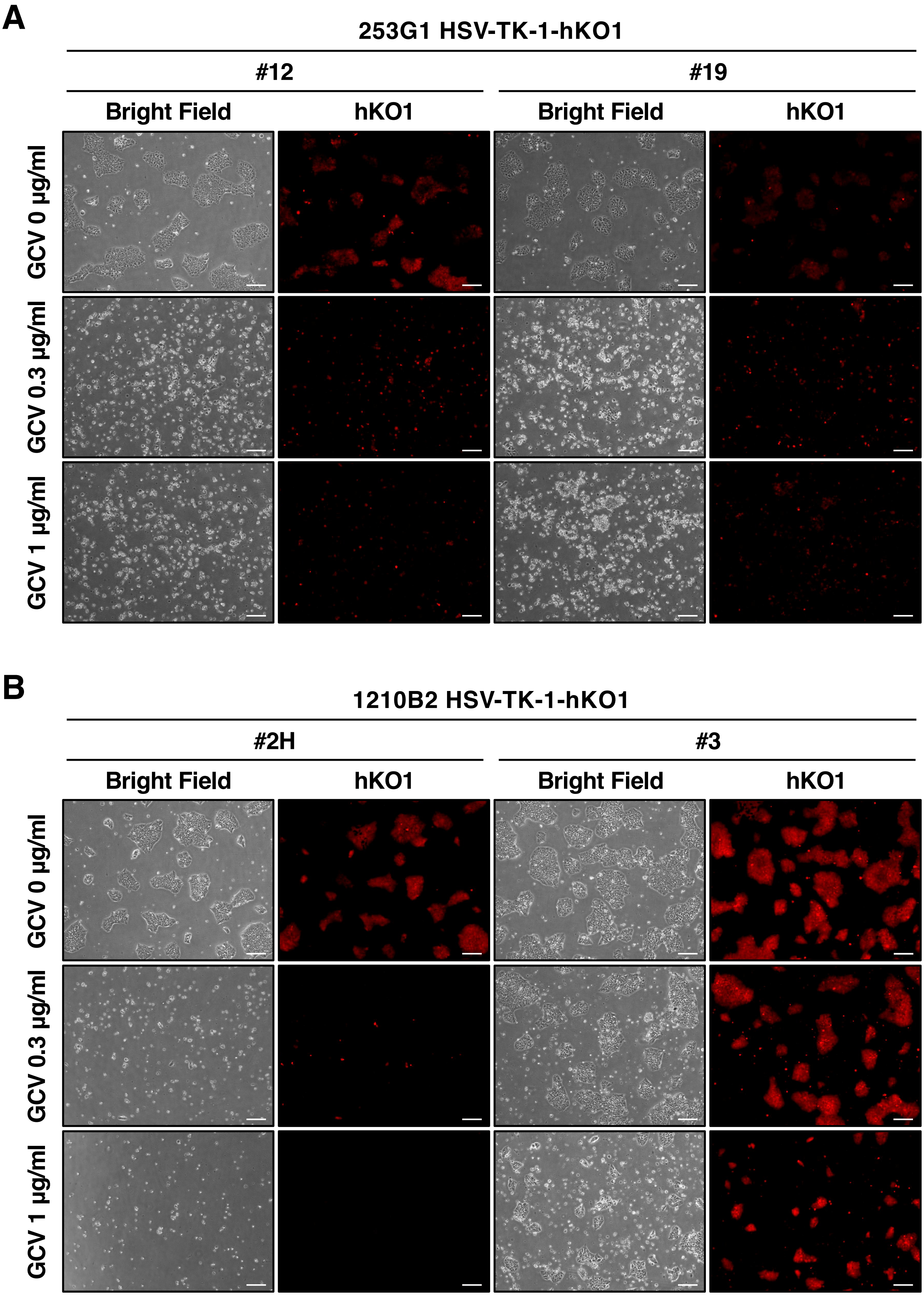

Supplementary Figure 2. GCV sensitivity of hKO1-positive iPSC clones transduced with the lentiviral vector CSII-EF-HSV-TK-1-IRES2-hKO1. (**A**–**B**) hKO1-positive iPSC clones, (**A**) 253G1 HSV-TK-1-hKO1 (#12, #19) and (**B**) 1210B2 HSV-TK-1-hKO1 (#2H, #3), were cultured in the presence or absence of GCV (0, 0.3, 1 μg/ml) for 3 days. Representative images show reduced viability with increasing GCV. Scale bar, 200μm.

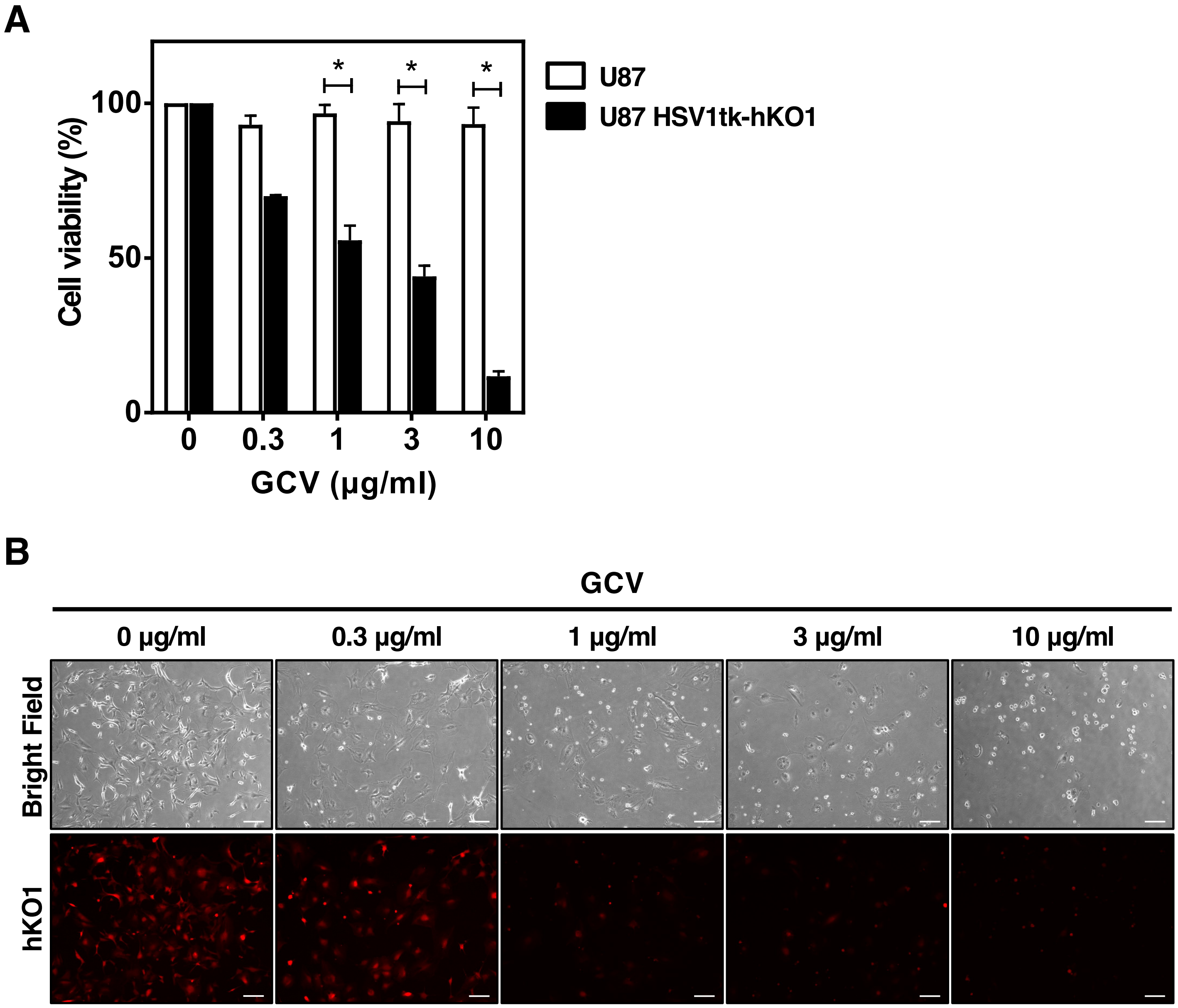

Supplementary Figure 3. GCV sensitivity of U87 human glioblastoma cells that expressed the *HSV1tk* gene. (**A**) U87 cells were transduced with the lentiviral vector CSII-EF-HSV1tk-IRES2-hKO1, and hKO1^{high} populations were FACS-sorted and were expanded. U87 and U87-HSV1tk-hKO1 cells were cultured in the presence of various concentrations of GCV for 3 days. Cell viability was assessed by the CCK-8 assay. U87-HSV1tk-hKO1 cells showed sensitivity to GCV in a dose-dependent manner. The percent cell viability was calculated relative to non-transduced U87 cells in the absence of GCV. Data represent the mean ± SEM (n = 4). *, p <0.05. (**B**) Representative images of U87-HSV1tk-hKO1 cells cultured with various GCV concentrations (0, 0.3, 1, 3, 10 μg/ml) for 3 days. Scale bar, 200μm.

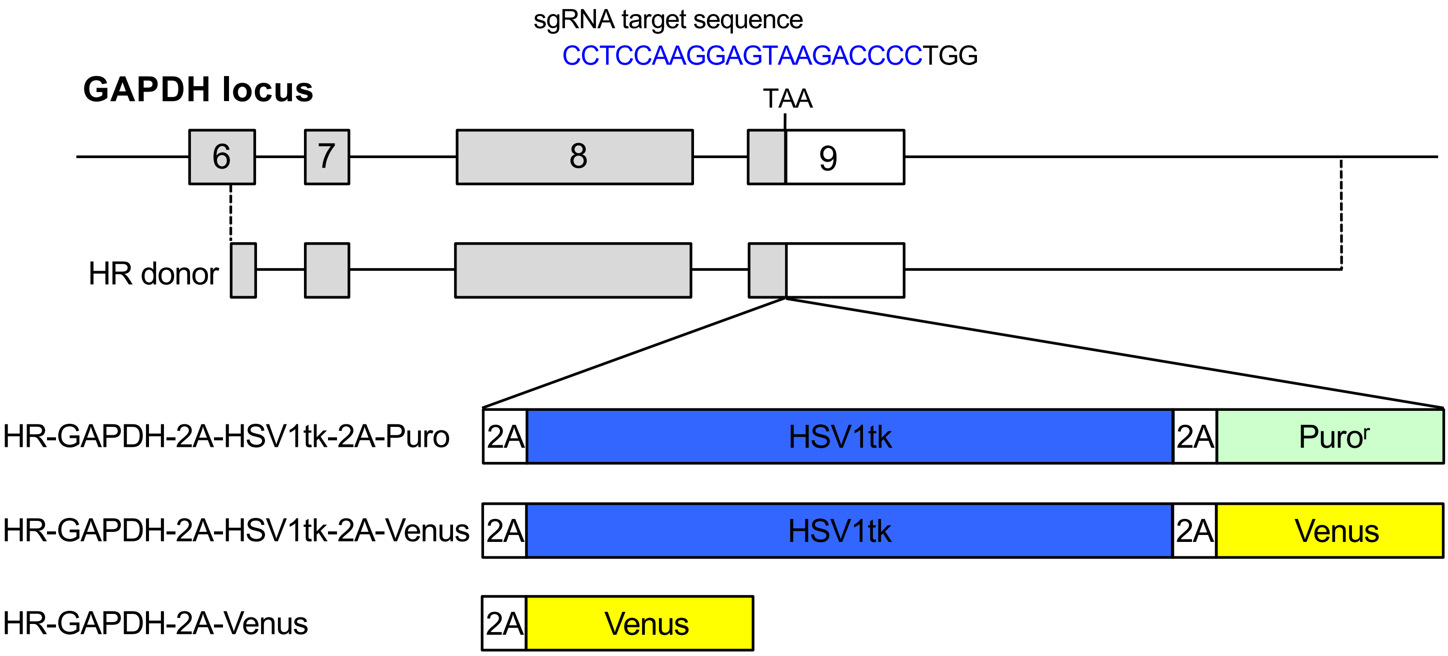

Supplementary Figure 4. Schematic depiction of the CRISPR/Cas9-mediated strategy for inserting the *HSV1tk* gene into the *GAPDH* locus. Human *GAPDH* exons are gray boxes, and the stop codon (TAA) is located in the exon 9. The single guide RNA (sgRNA) target sequence is shown in blue. The HR donor constructs are shown below.

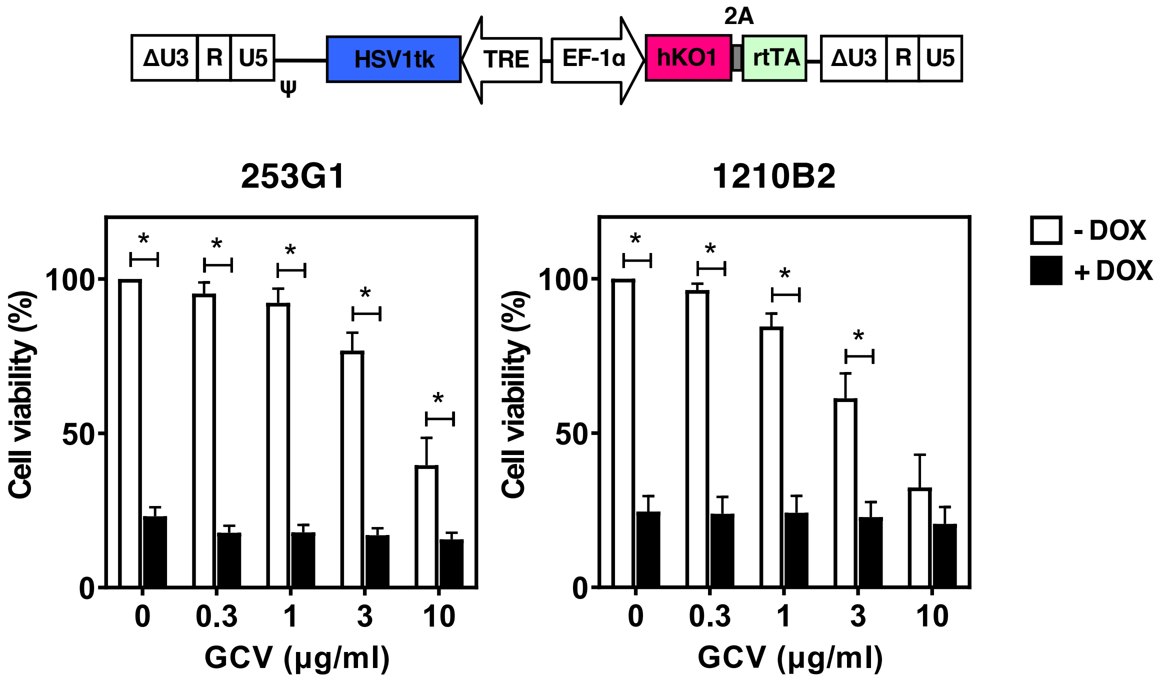

Supplementary Figure 5. Cytotoxicity of HSV-TK expression with the Tet-inducible HSV1tk lentiviral vector. Schematic representations of the integrated proviral form of the Tet-inducible lentiviral vector carrying the *HSV1tk* gene. TRE, Tet-responsive promoter; rtTA; reverse Tet-controlled transactivator protein gene. 253G1 iPSCs and 1210B2 iPSCs transduced with the Tet-inducible lentiviral vector carrying the *HSV1tk* gene were cultured in the presence of various concentrations of GCV, with or without 1 μg/ml doxycycline (Dox) for 2–3 days. Cell viability was assessed by the CCK-8 assay. The percent cell viability was calculated relative to cells in the absence of GCV without Dox. There was no significant difference in the results obtained on days 2 and 3 of culture. Data represent the mean ± SEM (n = 4). *, p <0.05.

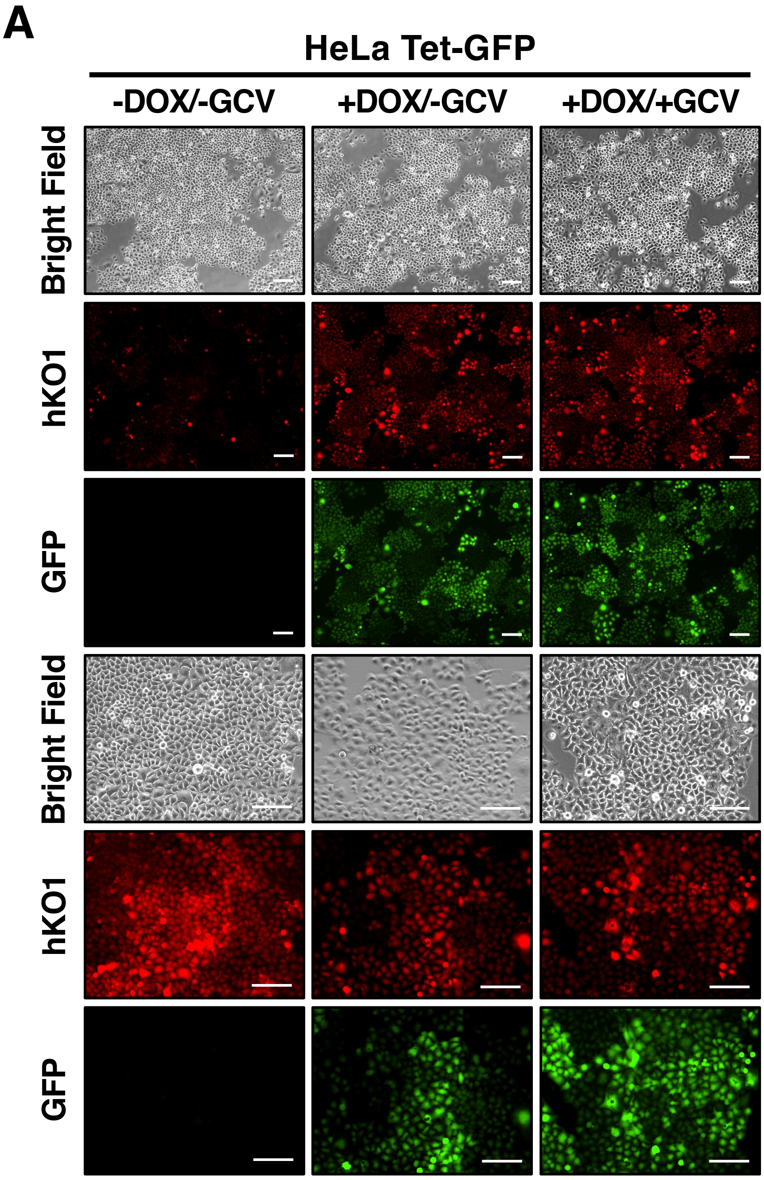

Supplementary Figure 6. Cytotoxicity of HSV-TK expression in HeLa cells. HeLa cells were transduced with Tet-inducible lentiviral vectors carrying (**A**) the *GFP* gene, (**B**) the *HSV-TK-1* gene, and (**C**) the *del-HSV-TK-1* gene were cultured in the presence (+) or absence (–) of 1 μg/ml GCV and/or 1 μg/ml Dox for 5 days. Representative images show cell viability (bright field), hKO1 expression (red), and GFP expression (green). Scale bar, 200μm.

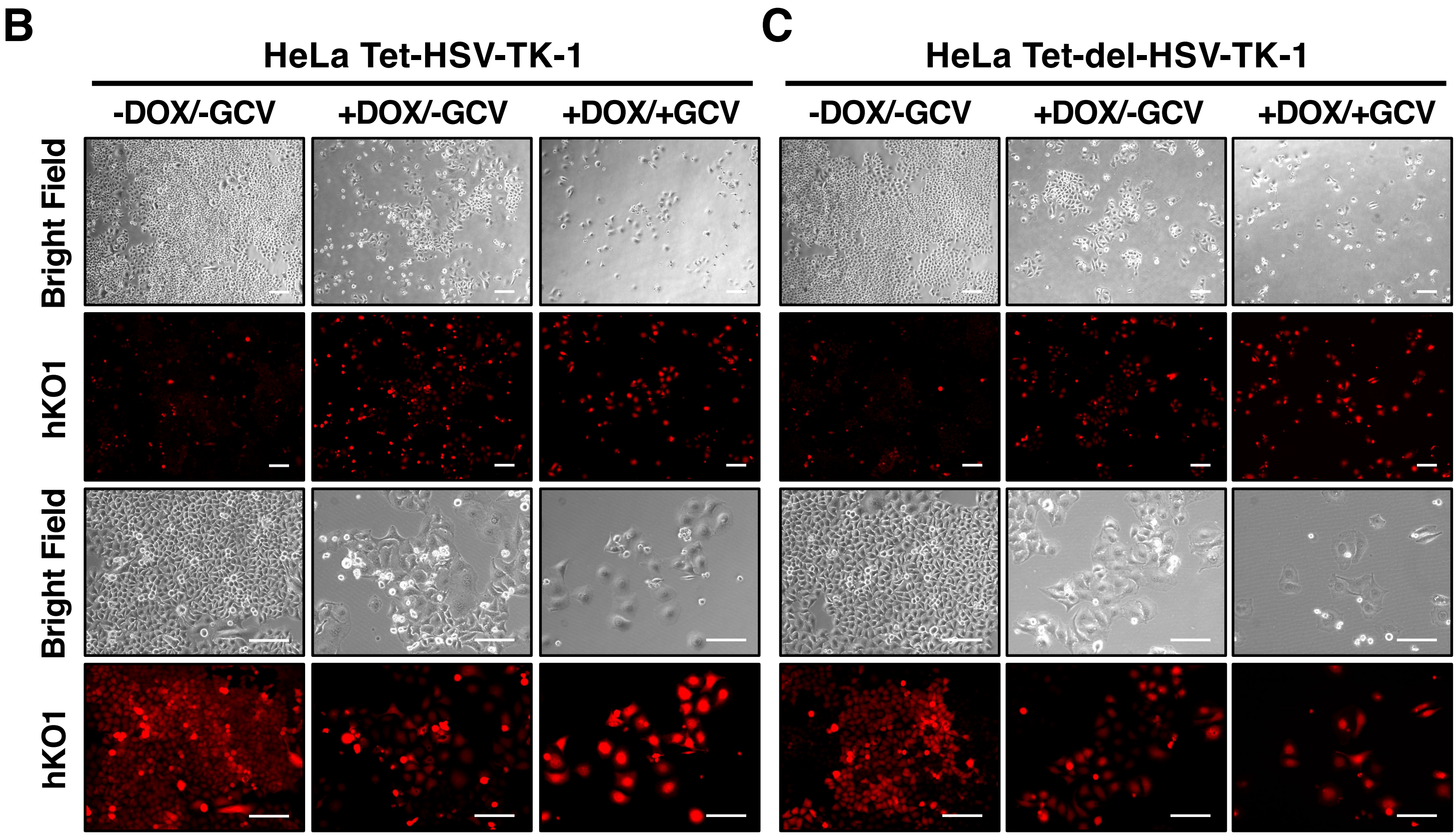

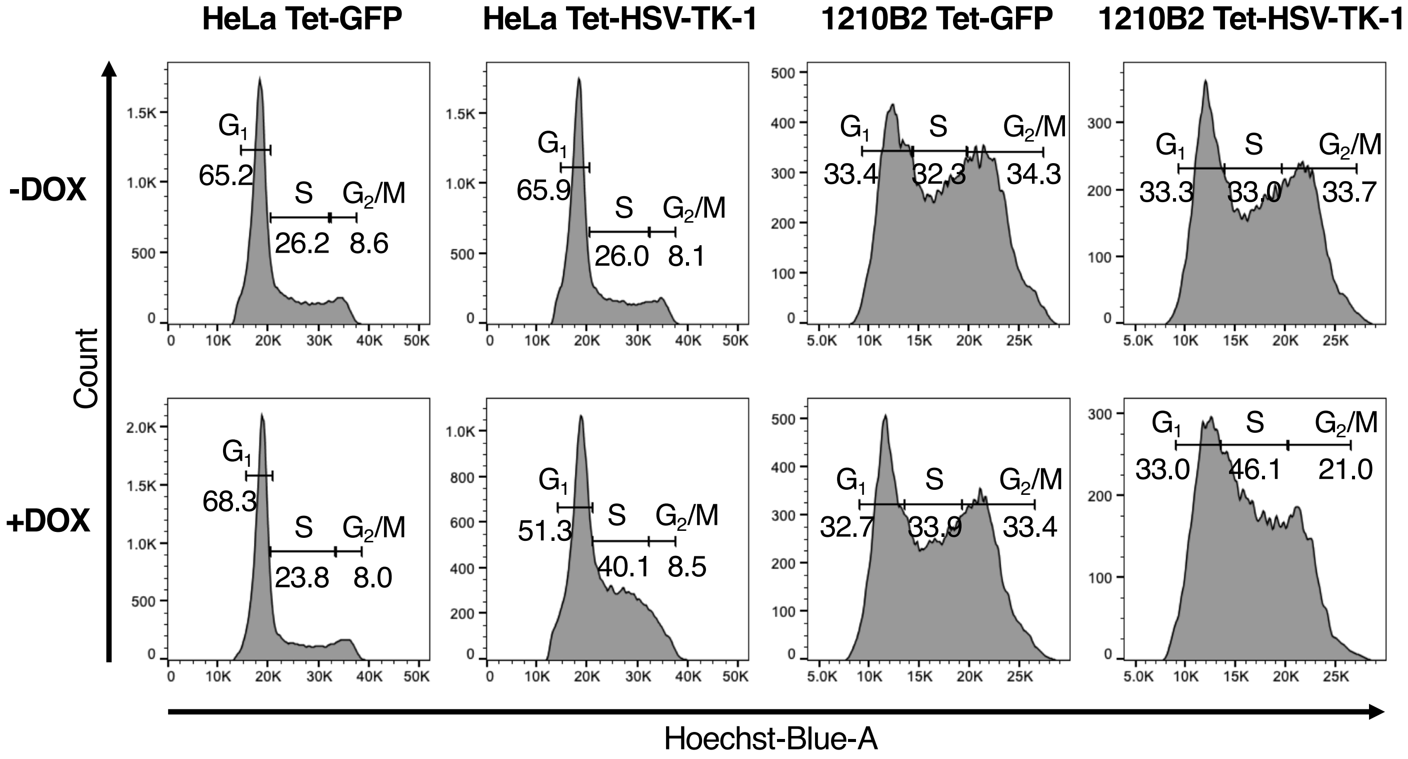

Supplementary Figure 7. Cell cycle analysis of human iPSCs and HeLa cells with Tet-inducible HSV-TK expression. 1210B2 iPSCs and HeLa cells transduced with the Tet-inducible lentiviral vectors carrying the *GFP* gene and the *HSV-TK-1* gene were FACS-sorted with high hKO1 expression levels and were cultured in the presence (+) or absence (–) of 1 µg/ml Dox for 2 days. Cells were stained with 5 µg/ml Hoechst 33342 for 30 min at 37˚C and cell cycle was analyzed with a FACSAria III and FlowJo software (BD Biosciences). Representative FACS histograms showing DNA content and percentage of cells in the G_1, S, and G_2/M phases are shown.

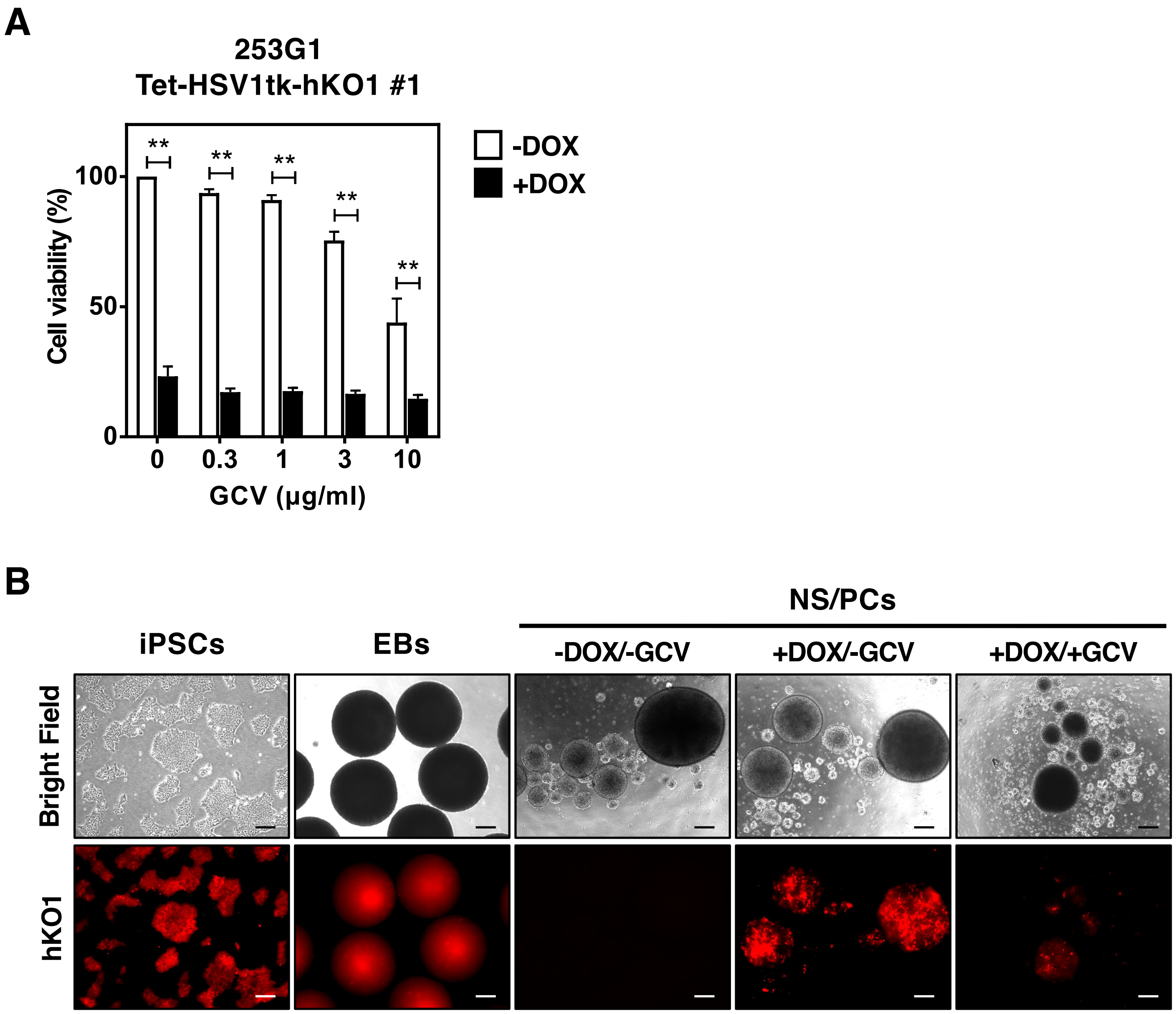

Supplementary Figure 8. Cytotoxicity of Tet-inducible HSV-TK expression in human iPSCs. (**A**) Single 253G1 iPSCs transduced with the Tet-inducible HSV1tk lentiviral vector were FACS-sorted with high hKO1 expression levels, and several hKO1-positive iPSC clones were established. One clone, 253G1 Tet-HSV1tk-hKO1 (#1), was cultured in the presence of various concentrations of GCV, with or without 1 μg/ml doxycycline (Dox) for 3 days. Cell viability was assessed by the CCK-8 assay. The percent cell viability was calculated relative to cells in the absence of GCV without Dox. Data represent the mean ± SEM (n = 5). **, *p* <0.01. (**B**) Representative images of 253G1 Tet-HSV1tk-hKO1 (#1) iPSCs, EBs, and NS/PCs. NS/PCs were cultured in the presence (+) or absence (–) of 1 μg/ml GCV and/or 1 μg/ml Dox for 7 days. Scale bar, 200μm.

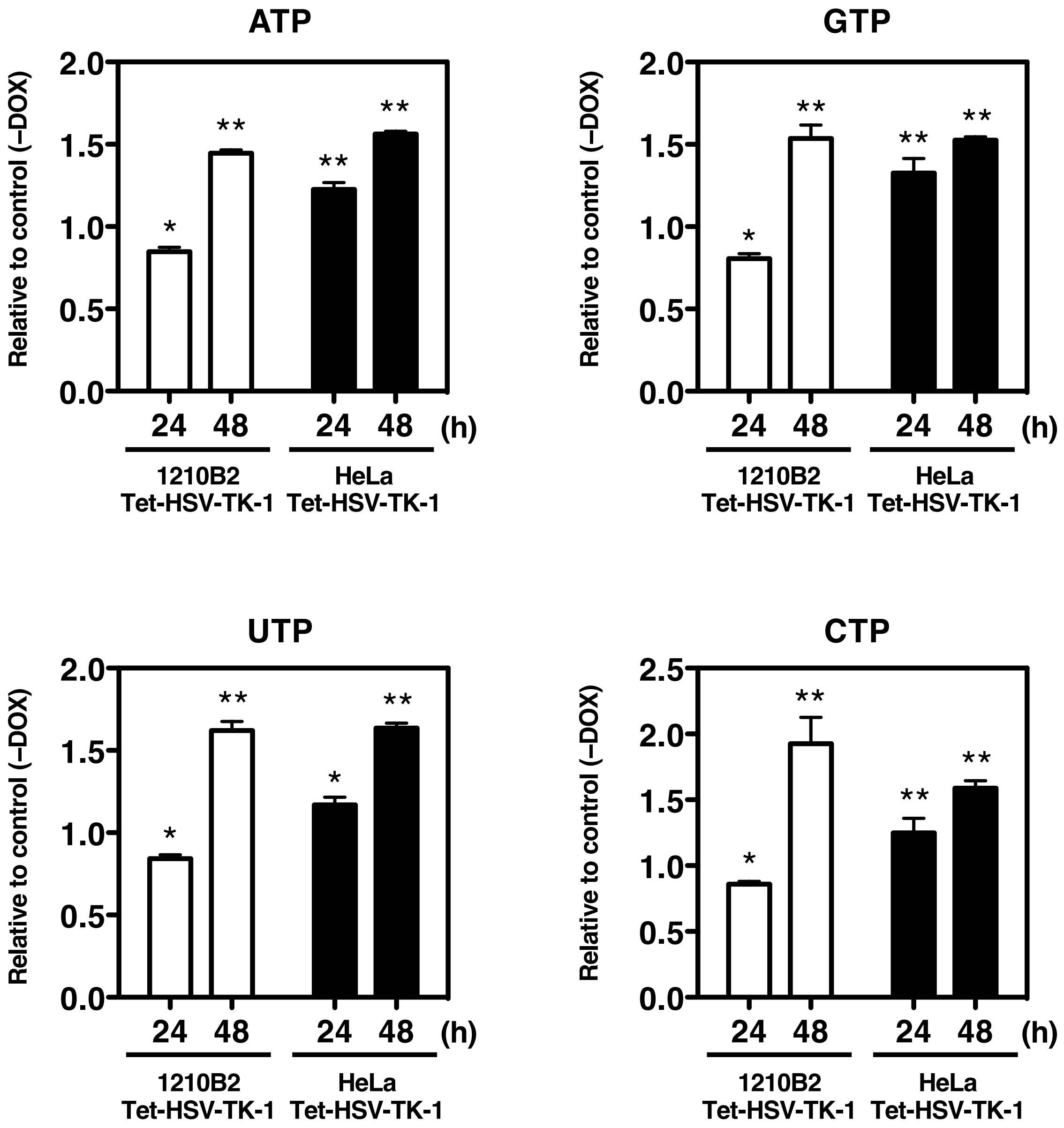

Supplementary Figure 9. NTP levels after HSV-TK expression in human iPSCs and HeLa cells. 1210B2 iPSCs and HeLa cells transduced with the Tet-inducible lentiviral vector carrying the *HSV-TK-1* gene were cultured with or without 1 μg/ml Dox. Metabolome analysis of the indicated nucleotides was performed at the indicated time points. Data are expressed as the fold change in nucleotide levels relative to corresponding cells without Dox (control). Data represent the mean ± SEM (n = 4–5). *, p <0.05; **, p <0.01.
